# Mastoid Obliteration Using S53P4 Bioactive Glass in Cholesteatoma Surgery: A 10-Year Single-Center Experience in 173 Adult Patients with Long-Term Magnetic Resonance Imaging Controlled Follow-up

**DOI:** 10.1097/MAO.0000000000003685

**Published:** 2022-09-13

**Authors:** Victor J. Kroon, Steven W. Mes, Pepijn A. Borggreven, Rick van de Langenberg, David R. Colnot, Jasper J. Quak

**Affiliations:** ∗ Department of Otolaryngology and Head and Neck Surgery, Diakonessenhuis Utrecht; † Faculty of Medicine, University Medical Center Utrecht, Utrecht, The Netherlands

**Keywords:** Bioactive glass, Cholesteatoma, Mastoid obliteration, S53P4, Surgery

## Abstract

To present the long-term outcomes of mastoid obliteration in cholesteatoma surgery using S53P4 bioactive glass (BAG) in an adult population.

**Study Design:**

Retrospective cohort study.

**Setting:**

Single-center study.

**Patients:**

All 173 adult patients who underwent primary or revision surgery for cholesteatoma with mastoid obliteration using S53P4 BAG with at least 1 year of follow-up including nonecho planar diffusion-weighted magnetic resonance imaging (MRI) (non-EP DWI MRI) and/or second-look surgery to evaluate recidivism. Both canal wall up (CWU) and canal wall down (CWD) procedures were included.

**Intervention(s):**

Patients underwent CWU or CWD mastoidectomy using S53P4 BAG.

**Main Outcome and Measures:**

Cholesteatoma recidivism, postoperative complications, Merchant grade, hearing outcome.

**Results:**

Cholesteatoma recidivism was assessed by MRI in 97% of all cases and second-look surgery look surgery in 3% of cases. After a mean follow-up period of 53 months, cholesteatoma recidivism was seen in 10% of the cases (n = 18). Using the Kaplan-Meier curve to extrapolate, a 5-year recidivism rate of 12% was estimated. Only minor complications occurred, all resolving spontaneously or after minor treatment. Merchant grade of 0 to 1 was achieved 95% of the patients, no persistently wet ears were observed. Closure of the air-bone gap within 20 dB was possible in 32%.

**Conclusion:**

In this long-term (up to 10 yr) follow-up study, we demonstrated the safety of S53P4 BAG. Minimal and only minor postoperative complications were observed. The effectiveness of BAG was indicated by the low rate of recidivism, even when using non-EP DWI MRI, a sensitive and specific noninvasive technique to detect cholesteatoma recidivism.

## INTRODUCTION

Cholesteatoma is a challenging disease involving the middle ear and mastoid, based on intrusion of stratified squamous epithelium, which can lead to the destruction of the ossicular chain and inner ear structures. The two main surgical procedures available for treatment of cholesteatoma are canal wall up (CWU), with preservation of the posterior wall of the external ear canal, and canal wall down (CWD), both in combination with a mastoidectomy. In more recent years, obliteration of the created mastoid cavity has gained in popularity, especially since the development of the non–echo-planar diffusion-weighted magnetic resonance imaging (non-EP DWI MRI) to detect cholesteatoma recidivism (1–3).

The obliteration technique has shown promising results, with similar, if not better, recidivism rates when compared with nonobliterated cholesteatoma ears (4,5). Currently, however, no Level 1 evidence is available and definitive conclusions cannot be made. There are multiple characteristics of obliteration to be considered. First, the obliteration technique reduces the total volume of the middle ear and mastoid, reducing the mucosal surface and the amount of gas exchange, thereby reducing gas absorption and normalizing middle ear pressure. Second, it serves as an anatomical barrier preventing new pocket formation to evolve to cholesteatoma. Third, in difficult cases, one can use the advantages of the CWD technique and obliterate the mastoid and reconstruct the posterior canal wall, ending up with a CWU situation (1).

In 2011, our center was one of the first to adopt S53P4 bioactive glass (BAG) as the material of choice for the obliteration of mastoid cavities after CWU or CWD procedures, allowing for one-stage eradication of cholesteatoma. The name S53P4 refers to the composition of the BAG, namely 53% SiO_2_, 4% P_2_O_5,_ 23% Na_2_O, and 20% CaO (6). It has two main qualities: the inhibition of bacteria and the stimulation of bone growth with osteostimulation and osteoconduction. In vitro studies have confirmed this effect of inhibition on a variety of clinically relevant bacteria (7,8). Moreover, a recent in vitro study by Sarin et al. (9) has shown that the increase in pH possibly also has an inhibitory effect on keratinocyte growth, thus inhibiting cholesteatoma development. The osteostimulative and osteoconductive properties of S53P4 BAG are related to the formation of a hydroxyapatite layer, which also functions as scaffolding for the osteoblasts (10,11). Previous studies on S53P4 BAG, including a study by our center, have showed excellent results (12–14). However, long-term results of this S53P4 obliteration technique are currently still lacking.

This is the first study with long-term follow-up of S53P4 BAG for mastoid obliteration in cholesteatoma surgery in an MRI-controlled adult cohort, with as primary outcome cholesteatoma recidivism.

## MATERIALS AND METHODS

### Ethical Considerations

This retrospective cohort study was conducted at the Diakonessenhuis Utrecht, a secondary referral center in the Netherlands. The study was in accordance with the ethical standards of the hospital institutional review board (registration number W21.162) and the 1964 declaration of Helsinki. Formal consent is not required for this type of retrospective study. The BAG S53P4, produced by BonAlive Biomaterials Ltd. (Turku, Finland), has received clearance for clinical use by the CE (2006).

### Patient Characteristics

Eligible for inclusion were all adult (≥18 yr) patients with cholesteatoma who underwent primary or revision single stage tympanomastoidectomy with mastoid obliteration using S53P4 BAG in the Diakonessenhuis Utrecht in the period 2011 to January 2021. Cholesteatoma was defined as a mass formed by keratinizing squamous epithelium in the tympanic or mastoid area, in accordance with the EAONO/JOS Joint Consensus Statement (15). Because of the retrospective character of this study, it was not feasible to use a cholesteatoma or middle ear grading system, as this information could not be obtained from the patient records. Keratin masses presenting in a preexisting radical cavity and could relieve spontaneously or by cleaning at the outpatient clinic were not considered cholesteatoma. Other inclusion criteria were a minimum follow-up period of 1 year postoperatively and the use of either non-EP DWI MRI and/or second-look surgery to evaluate recidivism. Patients were excluded if no non-EP DWI MRI or second look was performed postoperatively and if previous obliteration had been performed.

### Surgical Technique

Both CWU and CWD tympanomastoidectomy were evaluated, the surgical approach chosen depended on the surgeon's assessment. Under general anesthesia, a retroauricular skin incision was made to perform an extensive mastoidectomy. All cholesteatoma was subsequently removed from the middle ear and mastoid. Reconstruction of ossicular chain was performed if considered feasible. In CWU procedure, the posterior wall of the external ear canal was preserved, and the antrum was closed with tragus or concha cartilage or, if malleus head and incus were removed, cartilage was placed at the border of the mesotympanum and epitympanum. In CWD procedure, the posterior wall of the external ear canal was reconstructed with cartilaginous material. The mastoid cavity, and epitympanum in case of removal of the malleus head and incus or CWD procedure, was obliterated with S53P4 BAG granules moistened with 0.9% sterile saline solution. Musculoperiosteal vascularized flaps were used to cover and close the obliterated cavity. In general, tragal cartilage was used for tympanoplasty. Both CWU and CWD procedures were administered 2000 mg cefazolin intravenous intraoperatively and additionally, only after CWU procedures, a passive drain in the obliterated cavity and amoxicillin/clavulanic acid 3 to 5 days postoperatively.

### Variables and Clinical Outcome

Follow-up consisted of clinical otoscopy at 1 and 8 weeks after surgery, 3- to 6-month intervals for 1 year and, thereafter, once yearly unless more frequent visits were deemed necessary. A non-EP DWI MRI was made at 1, 3 and 5 years after surgery, or more frequent if deemed necessary by the treating physician. The primary outcome was cholesteatoma recidivism, which was defined as a mass of keratinizing squamous epithelium invading the tympanic space and/or mastoid cavity, diagnosed by either non-EP DWI MRI or second-look look operation. Recidivism can be caused by residual disease, defined as cholesteatoma left behind during the first surgery, or recurrent disease, defined as the development of a new retraction pocket with retention of keratinizing squamous epithelium. Secondary outcome parameters were as follows: procedure safety, Merchant grade at the most recent out-patient visit and audiometric performance. Procedure safety was defined as the absence of perioperative and postoperative complications up to 1 year after surgery. Merchant's grading system was used to evaluate postoperative otorrhea (16). Grades 0 to 1 were control of infection, Grades 2 to 3 were failure to control infection. Merchant grade was not scored in patients with cholesteatoma recidivism, as these patients may have presented with otorrhea as result of cholesteatoma recidivism and not of inflammation of the ear because of the surgical technique. Audiometric evaluation was performed preoperatively and postoperatively using pure-tone audiometry at 512, 1000, 2000, 4,000 Hz for both air and bone conduction (AC and BC, respectively), and the averages were calculated. The average air-bone gap (ABG) was calculated. Audiometric data were only analyzed if both preoperative and postoperative hearing tests were complete and performed within 6 months of surgery, including of patients without reconstruction of the ossicular chain.

### Statistical Methods and Data Analysis

Data were analyzed using SPSS statistics (version 27, IBM Corp, Armonk, NY). Kaplan-Meier Curve was used to extrapolate the 5-year recidivism rate, compensating for loss to follow-up and patients with a follow-up of less than 5 years. For audiometric evaluation, Wilcoxon signed-rank test was used for comparison of preoperative and postoperative AC, BC, and ABG. Mann-Whitney *U* test was used to compare postoperative ABG between different subsets of cases. AC, BC, and ABG preoperative and postoperative were presented as boxplots and scatterplots. *p* Value <0.05 was considered statically significant.

## RESULTS

In our hospital, we have performed a total of 789 mastoid obliterations using S53P4 BAG. In total, 223 surgeries with S53P4 BAG obliteration were performed for cholesteatoma in adult patients up to January 2021. Of these patients, 50 did not have adequate MRI-controlled or second-look surgery as follow-up. Reasons are summarized in Supplementary Table 1 (http://links.lww.com/MAO/B485).

In total, 173 cases (171 patients) were included, all operated in the period 2011 to 2021. The mean age at time of surgery was 42 years (standard deviation [SD], 16; range, 18–80 yr), 98 patients (57%) were men, and 77 cases (45%) were left ears (Table 1). Two patients had bilateral surgery for cholesteatoma. Eighty-five cases (49%) had previously been operated for cholesteatoma.

**TABLE 1:** Patient characteristics and surgical technique, n (%)

Total cases included	173 (100)
Male	98 (57)
Age (mean ± SD), yr	42 ± 16
Primary surgery	88 (51)
Side	
Left	77 (45)
Right	96 (55)
Location cholesteatoma	
Pars flaccida	84 (49)
Pars tensa	56 (32)
Combination tensa and flaccida	16 (9)
Residue after previous surgery	4 (2)
Unknown	13 (8)
Types of surgery	
CWU	36 (21)
CWD	137 (79)
Ossicular chain at the end of surgery	
Intact	35 (20)
Restored with cartilage	11 (8)
Incus interposition	8 (5)
PORP	74 (43)
TORP	31 (18)
Not repaired	13 (6)
Unknown	1 (1)

CWD indicates canal wall down; CWU, canal wall up; PORP, partial ossicular replacement prosthesis; TORP, total ossicular replacement prosthesis.

### Surgical Technique and Safety

CWU procedure was performed in 36 of 173 cases (21%) and CWD in 137 of 173 cases (79%). Of the CWD surgeries, 63 (46%) were primary CWD surgeries and 74 (54%) were secondary surgeries for recidivism after previous surgeries for cholesteatoma. Eleven of the CWU cases had previously been operated for cholesteatoma.

Reconstruction of the ossicular chain was performed in 124 cases (72%) (Table 1). Materials used for reconstruction were cartilage in 11 ears (6%), incus interposition in 8 ears (5%), titanium partial ossicular replacement prostheses in 74 ears (43%) and titanium total ossicular replacement prostheses in 31 ears (18%). In the remaining 49 cases, the ossicular chain was intact (20%), not reconstructed (6%) or unknown (1%).

Importantly, no patients were observed with major complications such as damage to the facial nerve or labyrinth. Also, no damage to the inner ear or total deafness due to the surgery was observed. Minor postoperative complications were seen in 21 cases (12%), often being short-term mild otorrhea, which was easily treated with oral antibiotics (13 cases) (Table 2).

**TABLE 2:** Postoperative complications (n)

Minor Complications	
Mild otorrhea requiring oral antibiotics	13
Recurrent perforation of the tympanic membrane	3
Acute otitis media	2
Otitis media with effusion	1
Retroauricular wound infection requiring oral antibiotics	1
Allergic reaction to antibiotics	1
Total	21

### Cholesteatoma Recidivism

**FIG. 1:**
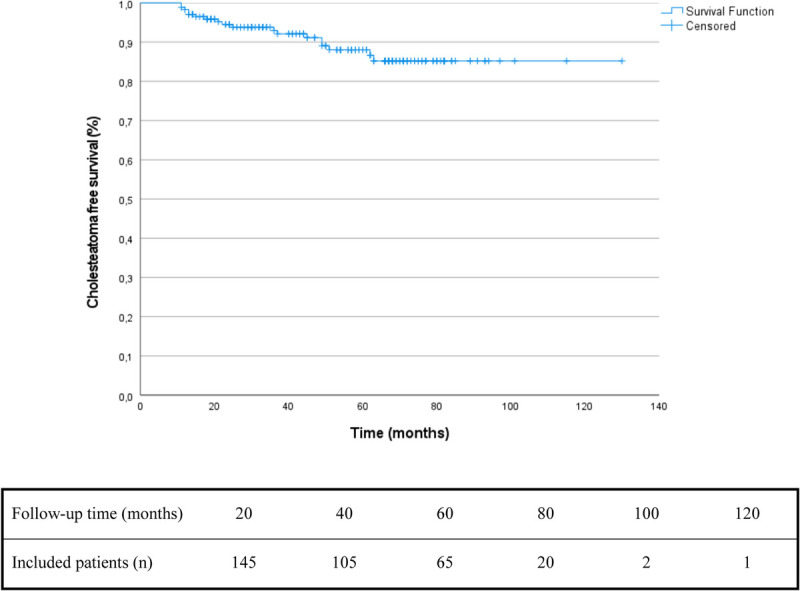
Kaplan-Meier curve to estimate cholesteatoma free survival, indicating 12% recidivism at 5 years following surgery.

The mean follow-up period postoperatively for the 173 included cases was 53 months (S.D., 26 mo; range, 12–130 mo). Non-EP DWI MRI or second-look surgery were used for the detection of recidivism with a mean follow-up period until the last MRI of 43 months (SD, 25 mo; range, 5–122 mo). In total, 361 MRI scans were made in 167 cases (97%, average of 2.1 per case), the remaining six cases (3%) underwent second-look surgeries solely, often with the main goal to further improve the hearing status. A total of 18 cases (10%) developed cholesteatoma recidivism with a mean time to recidivism of 31 months (SD, 18 mo, range, 11–63 mo), 11 being recurrences and seven being residual cholesteatoma. The Kaplan-Meier cure estimated a recidivism rate of 12% at 5 years after surgery (Fig. 1). The cholesteatoma was detected by MRI in 15 cases (83%). The others were detected by otoscopic examination (n = 1) or second-look surgery (n = 2). Recidivism locations were epitympanum (n = 7), mesotympanum (n = 3), tympanic sinus (n = 6), stapes footplate (n = 2). No cholesteatoma involving the obliterated cavity was observed. Three recidivisms were diagnosed in the first year after surgery, six in the second year, two in the third year, two in the fourth year, three in the fifth year and two were diagnosed more than 5 years after surgery. Only one recidivism was observed in the CWU group, demonstrating a recidivism rate of 3%. The other 17 were seen after CWD procedures, resulting in a recidivism rate of 12%. Further details are displayed in Supplementary Table 2 (http://links.lww.com/MAO/B485).

Surgical revision techniques to treat cholesteatoma recidivism were CWD (15/18, 83%), CWU (1/18, 6%), endoscopic transcanal (1/18, 6%), and one patient is awaiting revision surgery at time of publication. The BAG granules were easy to remove if needed during revision surgery and anatomic structures could readily be recognized. Mastoid cavity was reobliterated if BAG granules were removed intraoperatively for access to the middle ear. One patient developed a second recidivism 36 months after his first revision, requiring a second revision CWD procedure. Two patients received revision surgery in other hospitals, therefore being lost to follow-up. The other 14 revision patients remained cholesteatoma free with an average follow-up period postrevision surgery of 39 months (SD, 26 mo; range, 1–85 mo).

### Merchant Grade and Audiometric Evaluation

At the most recent out-patient visit, control of the infection (Merchant Grades 0–1) was achieved in 148 cases (95%) and no control of the infection (Merchant Grade 2–3) in 7 cases (5%), all being Merchant Grade 2.

**FIG. 2:**
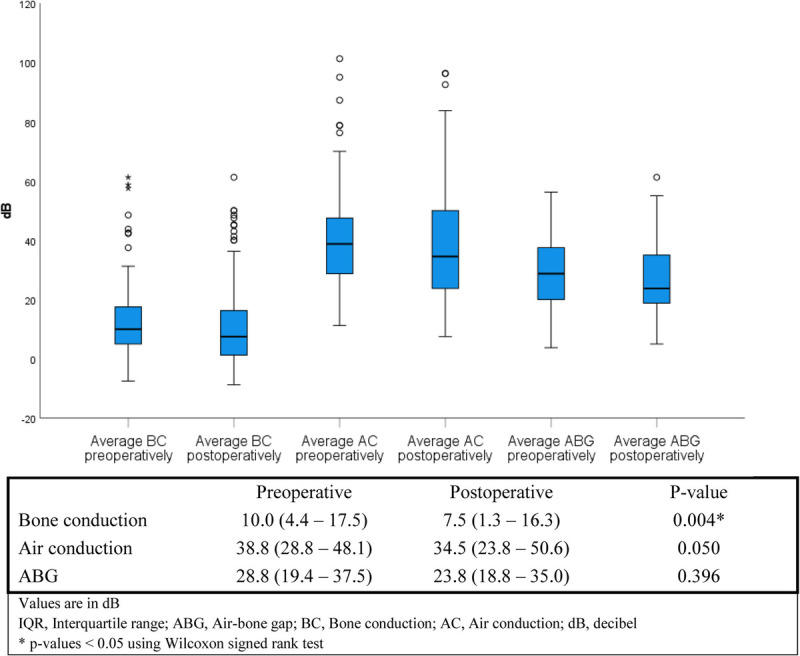
Boxplots and table of audiometric evaluation for all patients with complete audiometry (median, IQR).

**FIG. 3:**
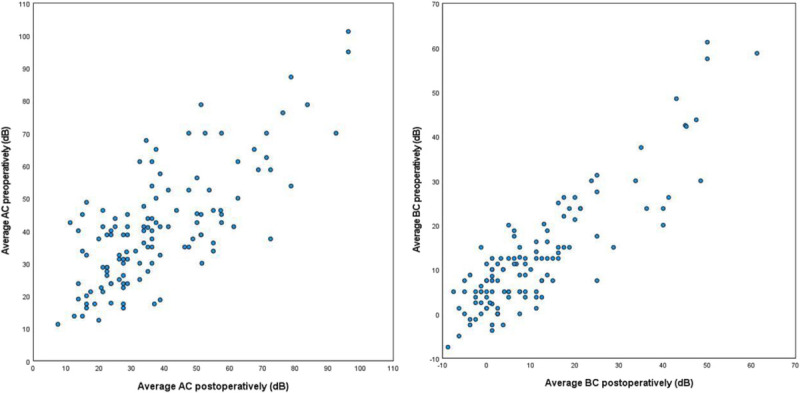
Scatterplots representing the change of average AC and BC from preoperatively to postoperatively, dots represent individual patients.

**FIG. 4:**
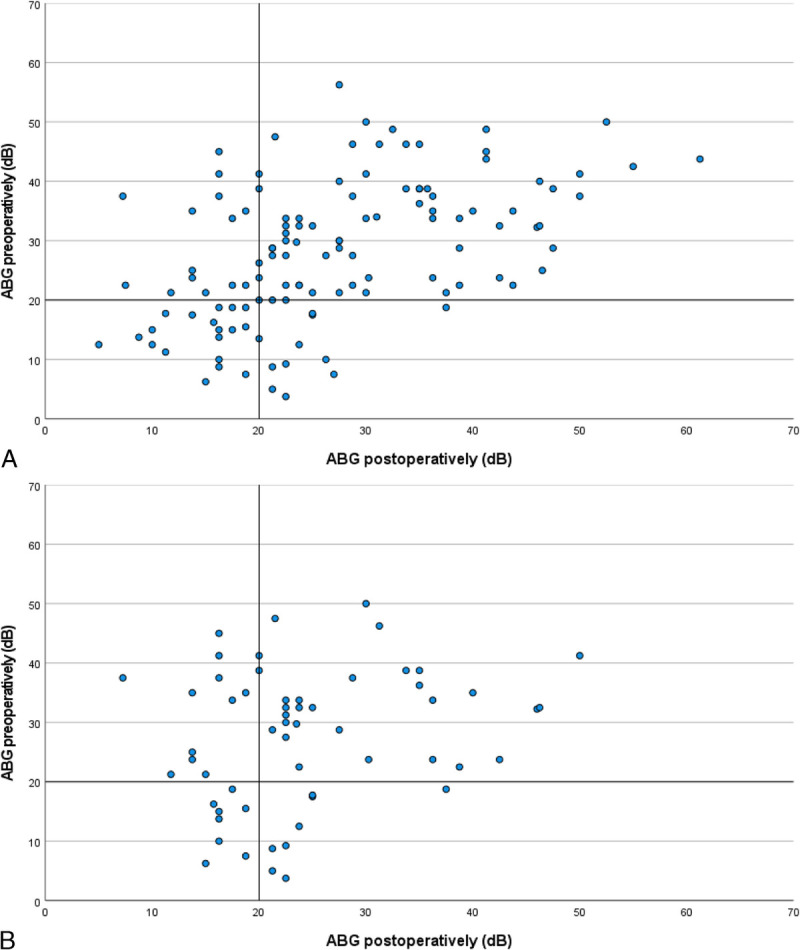
Scatter plots comparing the preoperative ABG to the postoperative ABG. The horizontal line indicates preoperative ABG = 20 dB, the vertical line indicates postoperative ABG = 20 dB. Points in the left-under or right-upper quadrant did not change pre- to postoperatively. Left-upper quadrant improved postoperatively and right-under quadrant deteriorated postoperatively. *A,* All patients. *B,* Subgroup of patients with ossicular reconstruction using a PORP.

Complete audiometric evaluation was available for 121 cases (70%). AC did not improve significantly after surgery (38.8 dB; interquartile range [IQR], 28.8–48.1 dB versus 34.5 dB; IQR, 23.8–50.6 dB, respectively, *p* = 0.050; Fig. 2). Bone conduction did not deteriorate after mastoid obliteration with S53P4 BAG, thus showing no signs of inner ear toxicity of the BAG. Scatterplots represent the preoperative and postoperative AC and BC of individual patients (Fig. 3). The ABG did not change significantly (28.8 dB; IQR, 19.4–37.5 versus 23.8 dB; IQR, 18.8–35.0; *p* = 0.396; Fig. 2). Subset analyses of the patients with CWU or CWD procedures or partial ossicular replacement prosthesis (PORP) or total ossicular replacement prosthesis (TORP) reconstruction also showed no significant improvement in ABG preoperatively to postoperatively. However, comparing the postoperative ABG of PORP to TORP showed a significantly improved outcome for PORP group (median ABG, 22.5 dB; IQR, 16.9–30.1 dB versus 34.4 dB; IQR, 26.6–43.4 dB, respectively; *p* < 0.001 and median AC, 33.8 dB; IQR, 22.5–47.5 dB versus 45 dB; IQR, 27.5–65.9 dB, respectively; *p* = 0.013). The CWU group also performed significantly better postoperatively than the CWD group (median ABG 22.5 dB IQR 16.3–27.5 versus 26.6 dB; IQR, 19.7–36.3 dB, respectively; *p* = 0.033), but the clinical significance of this difference is unknown. Closure of the ABG to ≤20 dB was achieved in 39 cases (32%; Fig. 4A). In the subset patients with a PORP reconstruction, closure of the ABG was achieved in 21 cases (37%; Fig. 4B).

## DISCUSSION

In this retrospective cohort study of 173 adult patients, we show that single-stage mastoid obliteration using S53P4 BAG in either CWU or CWD procedure for cholesteatomas is safe and effective. Only minor complications, such as otorrhea and/or middle ear infection, were observed in 21 cases (13%), and all resolved spontaneously or after local or systemic treatment. Recidivism rates were 10% after a mean follow-up period of 53 months and an estimated 12% after 5 years with extrapolation on the Kaplan-Meier curve. Control of infection (Merchant Grade 0–1) was achieved in 95% of the patients without cholesteatoma recidivism at the latest out-patient visit. Postoperative audiometry showed no decline in patient’s hearing status. The main strengths of this study are the size, the homogeneity regarding diagnosis and age, that all patients have received either non-EP DWI MRI (167/173, 97%) or a second look surgery (6/173, 3%) to assess recidivism and the length of follow-up.

BAG as obliteration material has benefit of preservation of volume over time, unlimited availability, not having to be impregnated in antibiotics intraoperatively and no possibility of donor site morbidity (17–19). Another important quality of BAG that we noticed was how effortless removal was during revision surgery. This allowed for landmark recognition and, therefore, safe and efficient surgery. Also important, we never observed residual disease in the obliterated cavity, which is in line with other publications (12).

Little studies were found comparing different materials for obliteration. A review of synthetic materials by Skoulakis et al. (20) deemed BAG the most reliable material. A recent study by Mishra et al. (21), comparing bone pâté to BAG, showed no significant differences in recidivism or complications. Long-term outcomes in a small cohort showed that obliteration with hydroxyapatite resulted in high rates of revision surgery and recidivism (22). Further research is needed to properly compare different obliteration materials.

There are also some potential disadvantages of obliteration to be pointed out, such as the extra cost of the obliteration material, the theoretical additional operative time needed to obliterate the mastoid cavity and the need for imaging. Therefore, future research should also investigate the costs and cost-effectiveness of obliterative techniques.

Since 2012, numerous studies have been published on the use of S53P4 BAG for mastoid obliteration, but long-term follow-up after cholesteatoma surgery is lacking (12–14,23,24). Our study shows a recidivism rate of 10% after a mean follow-up period of 53 months, which is in line with our initial publication with short-term results by de Veij Mestdagh et al (12). Our goal was to demonstrate the safety of S53P4 BAG and create a realistic view of the efficacy. We included all adults with cholesteatoma and adequate follow-up who underwent mastoid obliteration using S53P4 BAG in our center, including both CWU and CWD and both primary and revision surgery. Interestingly, only one case of cholesteatoma recidivism was detected in the CWU group, resulting in a recidivism rate of only 3%. It suggests that in a selected cohort, most likely with limited or accessible disease, CWU is the treatment strategy of choice.

There are several reference studies to compare our findings with. A systematic review comparing CWU versus CWD versus CWD with reconstruction by Harris et al. (4) showed a recidivism rate in the reconstruction group of 5.3%. The systematic review by van der Toom et al. (5) found a recidivism rate of 10% in 1534 patients. These rates are both lower than our estimated recidivism rate. However, none of the studies included by Harris and only one of the studies included by van der Toom used non-EP DWI MRI to diagnose cholesteatoma recidivisms. Most often only a computed tomography (CT) scan was used, which has shown to have a sensitivity and specificity as low as 42.9% and 48.3%, respectively (25). The same problem arises in other studies reporting cholesteatoma recidivisms after obliteration, often using CT scans or only otoscopic examination to diagnose recidivism (26–30). Although second-look surgery is traditionally considered the “gold standard” in detecting cholesteatoma recidivism, imaging has the advantage of being noninvasive and therefore repeatable during follow-up without major impact on the patient. Lingam and Bassett (31) conducted a meta-analysis on the performance of non-EP DWI MRI in the detection of cholesteatoma with 1152 patients. They demonstrated a sensitivity of 0.91 and specificity of 0.92, thus showing superiority over CT scans to detect cholesteatoma. It also means that our study would be expected to find a higher rate of recidivism, as we use a more sensitive detection technique than CT or otoscopy. Eighty-four percent of our recidivism cases were detected by non-EP DWI MRI and would likely have been missed by otoscopic examination alone. It is currently unknown if all these cholesteatoma recidivisms would have become clinically relevant. A prospective study to evaluate a wait and see strategy, especially in cases with severely deteriorated hearing, is needed to assess the clinical relevancy of the detected recidivisms.

We found three studies to be fairly comparable to our study regarding follow-up protocol. First, van der Toom et al. (32) compared CWU with obliteration to CWU and CWD in a large study of 337 patients (208 obliteration patients), with an estimated 5-year recidivism rate of 7.6%. They used DWI-MRI to evaluate recidivism, but scans were only performed 1 and 5 years after surgery. As their mean follow-up time was 29.5 months, a fair part of their patients would not have had their 5-year MRI scan. This could cause an underestimation of recidivism by the Kaplan-Meier curve, as not enough events were registered. Second, van Waegeningh et al. (33) evaluating CWU with obliteration, showed a recidivism rate of 3%. This, however, was a reasonably small cohort of 61 cases, mainly primary surgery and all operated by a single surgeon in a tertiary referral center. In contrast, in our study, surgery was performed by four surgeons with a variation in number of operated ears and years of experience. Consequently, this might reflect more the real-world situation. Furthermore, no Kaplan-Meier curve was used to estimate the 5-year recidivism rate, limiting the study results. Third, the study most comparable to ours regarding follow-up protocol was published by Hellingman et al (34). They presented similar recidivism rates of 11% in the adult population. However, they only evaluated CWU procedures, the average follow-up time was shorter (39.6 months), and no Kaplan-Meier curve was used to estimate 5-year recidivism rate. Overall, we think that our results are realistic and competitive in comparison with the current available literature. Long-term follow-up with regular imaging is necessary to properly evaluate the efficacy of different treatment strategies for cholesteatoma. Based on our results, we advocate a follow-up regime of non-EP DWI MRI at 1, 3, and 5 years after surgery, to optimally detect recidivism cases. This is in line with a recent study, also advocating an MRI at 1, 3, and 5 years after surgery (35). If the postoperative hearing status is not deteriorated (ABG ≤ 20 dB), the frequency of non-EP DWI MRI can be increased to ensure that recidivisms are detected before stapes destruction has occurred (36).

We showed that our technique results in very few complications and that, when any were present, these complications mostly resolved spontaneously or by simple treatment. Furthermore, no patients with a Merchant score of 3, indicating persistent wet ear, were observed and control of the infection was achieved in 95% of the patients. This is especially important since most of our surgeries were CWD procedures, which historically more often result in persistently discharging cavities (37). It shows that clinicians should not be deterred to perform a CWD procedure if needed, as long as an adequate reconstruction is done. Audiometric evaluation showed no significant improvement in AC or ABG. This is in line with other recent publications, showing that hearing status will not or only marginally improve after surgery (32–34). It is important for the preoperative counseling of patients. The main goal of the primary surgery is to create a dry and safe ear. Hearing status will most likely only improve in a small group of cases and patients should be made known about this. Hearing status was not negatively influenced by the S53P4 BAG. The combination of a dry ear and no ototoxicity makes patients eligible for hearing aids.

Our study also meets some limitations that have to be addressed. First, the retrospective character of this study did not allow for classification of the cholesteatomas with systems such as STAMCO or CHOLE since the detailed information required for staging was not always present in the operation report. However, the prognostic value of such staging may be limited, as pointed out by Eggink et al (38). Second, we did not compare our results with a cohort without obliteration, as our surgical technique changed after 2011 and the historic cohorts were not comparable with regard to follow-up protocol. Third, follow-up time in our cohort was not homogenous. However, since the mean follow-up period was 53 months and we also used a Kaplan-Meier curve to estimate 5-year recidivism rates, we feel that this has not significantly influenced our results. Fourth, 22% of the operated patients could not be included in this study, mainly because of lost to follow-up, which reflects real-world practice.

Our study has shown that S53P4 BAG is safe and effective for mastoid obliteration in cholesteatoma surgery in adult patients. We were able to confirm the results from our initial study in a larger population with long-term follow-up. The qualities of S53P4 BAG as obliteration material, being antibacterial and anti-keratinocytes, easily removable, osteoconductive, osteostimulative, and the unlimited availability, are important benefits to consider.

Finally, the all-American invention of turning glass into bone by Professor Hench in the 1970s at last got its recognition by the FDA approval in February 2022, putatively awaiting an even wider clinical application (39).

## Supplementary Material

SUPPLEMENTARY MATERIAL
