# Supplementary material for: Mastoid Obliteration Using S53P4 Bioactive Glass in Cholesteatoma Surgery: A 10-Year Single-Center Experience in 173 Adult Patients with Long-Term Magnetic Resonance Imaging Controlled Follow-up
Source: Otol Neurotol. 2022 Sep 13;43(10):1181–8. doi: 10.1097/MAO.0000000000003685 (PMC9671595; doi:10.1097/MAO.0000000000003685)
Supplement: SUPPLEMENTARY MATERIAL [file on-43-1181-s002.docx]

| Supplementary table 1. Reasons for inadequate follow-up (n) | |
| --- | --- |
| Lost to follow-up | 23 |
| Receiving regular checkups but did not receive an MRI | 15 |
| Moved to a different region | 4 |
| Claustrophobia or comorbidity | 4 |
| Planned but not yet performed | 4 |
| Total | 50 |

| Supplementary Table 2. Characteristics of recurrent and residual cases | | | | | | | | | | | | |
| --- | --- | --- | --- | --- | --- | --- | --- | --- | --- | --- | --- | --- |
| Case # | Sex, age (years) | Previous surgery for cholesteatoma | Type of surgery | ABG after primary surgery (dB) | Time to recidivism (months) | Merchant score before revision | Recurrent / residual | Location | Detection method | Size recidivism on MRI (mm) | Type of revision surgery | Recidivism free period after revision (months) |
|  | M, 21 | Yes | CWD | 26,25 | 12 | 0 | Recurrent | Tympanic sinus | MRI | 11 | CWD | n.a. (loss to follow-up) |
|  | M, 26 | Yes | CWD | 47,5 | 37 | 0 | Residual | Stapes footplate | MRI | 8 | CWD | 65 |
|  | M, 21 | Yes | CWU | 42,5 | 21 | 0 | Residual | Epitympanum | MRI | 8 | CWU | 85 |
|  | F, 36 | Yes | CWD | 50 | 22 | 0 | Residual | Stapes footplate | MRI | 3 | CWD | 76 |
|  | F, 50 | Yes | CWD | n.a. | 45 | 0 | Residual | Epitympanum | MRI | 6 | CWD | 36 |
|  | M, 23 | No | CWD | 17,5 | 36 | 2 | Recurrent | Mesotympanum | Otoscopic examination | n.a. | CWD | 54 |
|  | M, 65 | Yes | CWD | n.a. | 63 | 0 | Recurrent | Tympanic sinus | MRI | 8 | CWD | 20 |
|  | M, 57 | Yes | CWD | 16,25 | 49 | 0 | Recurrent | Tympanic sinus | MRI | 3 | CWD | 34 |
|  | M, 44 | No | CWD | 42,5 | 15 | 0 | Recurrent | Tympanic sinus | Second look surgery | n.a. | CWD | n.a. (2^nd^ recidivism) |
|  | M, 20 | Yes | CWD | n.a. | 18 | 2 | Recurrent | Tympanic sinus | MRI | 10 | CWD | 60 |
|  | F, 27 | Yes | CWD | 30 | 13 | 0 | Recurrent | Epitympanum | MRI | 6 | CWD | 50 |
|  | M, 30 | No | CWD | 18,75 | 11 | 0 | Residue | Epitympanum | MRI | 9 | CWD | n.a. (loss to follow-up) |
|  | F, 37 | Yes | CWD | 17,5 | 51 | 0 | Recurrent | Mesotympanum | MRI | 5 | Endoscopic transcanal | 17 |
|  | F, 31 | No | CWD | 20 | 49 | 2 | Recurrent | Mesotympanum | MRI | 7 | CWD | 8 |
|  | M, 50 | Yes | CWD | n.a. | 62 | 1 | Recurrent | Epitympanum | MRI | 11 | To be operated | To be operated |
|  | M, 26 | Yes | CWD | 47,5 | 11 | 2 | Residual | Tympanic sinus | MRI | 8 | CWD | 26 |
|  | V, 19 | No | CWD | 22,5 | 25 | 1 | Recurrent | Epitympanum | MRI | 6 | CWD | 1 |
|  | V, 63 | No | CWD | 30,25 | 13 | 0 | Residual | Epitympanum | 2nd look surgery | n.a. | CWD | 12 |
